# Use of reactive hyperemia - peripheral arterial tonometry and circulating biological markers to predict outcomes in sepsis

**DOI:** 10.5935/0103-507X.20160072

**Published:** 2016

**Authors:** Vandack Nobre, Thiago Bragança Ataíde, Luisa Caldeira Brant, Clara Rodrigues Oliveira, Lucas Vieira Rodrigues, Antonio Luiz Pinho Ribeiro, Fernanda Barbosa Lopes, Ivan Euclides Saraiva, Marcus Vinícius Andrade

**Affiliations:** 1 Intensive Care Service, Hospital das Clínicas, Universidade Federal de Minas Gerais - Belo Horizonte (MG), Brazil.; 2 Postgraduate Program in Infectious Diseases and Tropical Medicine, Internal Medicine Department, Faculdade de Medicina, Universidade Federal de Minas Gerais - Belo Horizonte (MG), Brazil.; 3 Postgraduate Program in Adult Health, Internal Medicine Department, Faculdade de Medicina, Universidade Federal de Minas Gerais - Belo Horizonte (MG), Brazil.

**Keywords:** Sepsis/metabolism, Endothelial cells/metabolism, Biomarkers, Hyperemia, Manometry/methods, Prognosis, Sepse/metabolismo, Células endoteliais/metabolismo, Biomarcadores, Hiperemia, Manometria/métodos, Prognóstico

## Abstract

**Objective:**

To evaluate the usefulness and prognostic value of reactive hyperemia - peripheral arterial tonometry in patients with sepsis. Moreover, we investigated the association of reactive hyperemia - peripheral arterial tonometry results with serum levels of certain inflammatory molecules.

**Methods:**

Prospective study, conducted in an 18-bed mixed intensive care unit for adults. The exclusion criteria included severe immunosuppression or antibiotic therapy initiated more than 48 hours before assessment. We measured the reactive hyperemia - peripheral arterial tonometry on inclusion (day 1) and on day 3. Interleukin-6, interleukin-10, high-mobility group box 1 protein and soluble ST2 levels were measured in the blood obtained upon inclusion.

**Results:**

Seventeen of the 79 patients (21.6%) enrolled were determined to have reactive hyperemia - peripheral arterial tonometry signals considered technically unreliable and were excluded from the study. Thus, 62 patients were included in the final analysis, and they underwent a total of 95 reactive hyperemia - peripheral arterial tonometry exams within the first 48 hours after inclusion. The mean age was 51.5 (SD: 18.9), and 49 (62%) of the patients were male. Reactive hyperemia indexes from days 1 and 3 were not associated with vasopressor need, Sequential Organ Failure Assessment score, Acute Physiology and Chronic Health Evaluation II score, or 28-day mortality. Among the patients who died, compared with survivors, there was a significant increase in the day 3 reactive hyperemia index compared with day 1 (p = 0.045). There was a weak negative correlation between the day 1 reactive hyperemia - peripheral arterial tonometry index and the levels of high-mobility group box 1 protein (r = -0.287).

**Conclusion:**

Technical difficulties and the lack of clear associations between the exam results and clinical severity or outcomes strongly limits the utility of reactive hyperemia - peripheral arterial tonometry in septic patients admitted to the intensive care unit.

## Abstract (pt)

**Objetivo:**

Avaliar a utilidade e o valor prognóstico da tonometria arterial periférica - hiperemia reativa em pacientes com sepse, e investigar a associação dos resultados deste exame com os níveis séricos de algumas moléculas inflamatórias.

**Métodos:**

Estudo prospectivo, realizado em uma unidade de terapia intensiva para pacientes adultos com 18 leitos. Os critérios de exclusão foram imunossupressão grave ou tratamento com antibióticos iniciado mais de 48 horas antes da avaliação. Aplicamos o exame de tonometria arterial periférica - hiperemia reativa quando da inclusão (dia 1) e no dia 3. Avaliamos os níveis de interleucina 6, interleucina 10, proteínas do grupo 1 de mobilidade alta e de ST2 solúvel no sangue obtido quando da inclusão.

**Resultados:**

Dos 79 pacientes incluídos, 17 (21,6%) tiveram os sinais da tonometria arterial periférica - hiperemia reativa considerados tecnicamente não confiáveis, tendo sido excluídos do estudo. Assim, incluímos na análise final 62 pacientes, que foram submetidos a 95 exames de tonometria arterial periférica - hiperemia reativa dentro das primeiras 48 horas após sua inclusão. A média de idade foi de 51,5 (DP: 18,9), e 49 (62%) dos pacientes eram do sexo masculino. Os índices de hiperemia reativa dos dias 1 e 3 não se associaram com necessidade de vasopressores, SOFA, APACHE II ou mortalidade aos 28 dias. Dentre os pacientes que morreram, em comparação aos sobreviventes, houve aumento significante nos índices de hiperemia reativa no dia 3 em comparação ao dia 1 (p = 0,0045). Ocorreu fraca correlação negativa entre o índice obtido por tonometria arterial periférica - hiperemia reativa no dia 1 e os níveis de proteínas do grupo 1 de mobilidade alta (r = -0,287).

**Conclusão:**

Dificuldades técnicas e falta de associações claras dos resultados do exame com a gravidade clínica e com o desfecho foram fortes limitantes da utilidade do exame de tonometria arterial periférica - hiperemia reativa em pacientes sépticos admitidos à unidade de terapia intensiva.

## INTRODUCTION

Severe sepsis is a major consumer of critical care resources,^(1)^ with approximately 750,000 cases per year in the United States.^(2,3)^ Although the prognosis has improved during recent years, mortality related to sepsis remains elevated, reaching 40 - 50% when shock is present.^(4)^

It has been suggested that an exaggerated and generalized adaptive response is the basis for the endothelial dysfunction observed in sepsis.^(5)^ Previously considered a layer of cells that coated vessels that convey oxygen and nutrients to peripheral organs, the endothelium is now regarded as a highly active and multifunctional tissue that plays a pivotal role in host protection against pathogens.^(5)^ Disorders of the endothelium appear to be directly involved in the physiopathology of sepsis-related organ dysfunction, the hallmark of the severe forms of sepsis.^(6)^

Given that the endothelium has a paramount relevance in sepsis, one can conceive that studying endothelial function in septic patients can be potentially useful to improve the management of this deadly syndrome. Endothelial function can be assessed by different tools; for example, the vasomotor response to pharmacologic or mechanic stimuli can be evaluated through invasive vascular catheterization or non-invasive tests or by measuring circulating biomarkers that reflect endothelial activation.^(7)^ Reactive hyperemia - peripheral arterial tonometry (RH-PAT) is a non-invasive and user-independent technique used to measure endothelial function in the microvessels of hand digits.^(8)^ The RH-PAT tests the ability of the microcirculation to vasodilate in response to shear stress caused by the release of blood flow after a period of interruption (i.e., ischemia), a response that is dependent on the bioavailability of nitric oxide. Recently, RH-PAT results have been associated with disease severity in patients with sepsis.^(9)^

Herein, we sought to investigate the association between RH-PAT values and 28-day all-cause mortality in a group of septic patients admitted to an intensive care unit of a teaching hospital. We further evaluated the association between microvascular endothelial function, based on RH-PAT exams, and disease severity and mortality using circulating levels of five inflammatory biological markers. Finally, we aimed to describe the difficulties observed during the use of RH-PAT in the intensive care unit setting.

## METHODS

This study involved a branch of a cohort of septic patients and was conducted in a mixed 18-bed intensive care unit (ICU) at the *Hospital das Clínicas* of the *Universidade Federal de Minas Gerais* (HC-UFMG). The HC-UFMG has 506 active beds and is a regional reference for the care of patients with diseases of moderate and high complexity.

From October 2012 to October 2013, all adult patients (≥ 18 years) admitted to the ICU with suspected or confirmed severe sepsis or septic shock, as defined according to the Sepsis 2 Consensus,^(6)^ were assessed for potential eligibility. The exclusion criteria were as follows: (1) patients with more than 48 hours of antibiotic treatment; (2) patients with a known diagnosis of HIV infection with CD4+ lymphocytes below 200 cells/mm^3^; (3) patients with severe neutropenia (less than 500 cells/mL); (4) patients post-transplant of solid organs or bone marrow or being treated with immunosuppressive therapy; (5) patients who received more than 0.5mg/kg of prednisone or equivalent in the last two weeks; (6) patients under palliative care; and (7) patients who suffered multiple trauma, burns, or major surgery in the previous 5 days. Specifically, for the RH-PAT study, we excluded patients with a low platelet count (< 20,000/mm^3^), patients presenting with other severe coagulation disorders (e.g., INR > 5, aPPT > 120 sec) and non-sedated patients unable to cooperate with the procedure due to agitation.

Patient data were prospectively collected using a dedicated case report form by consulting electronic and printed records. Data collection was performed by two physicians (TA and IS) and confirmed by two team managers (CRO and LB). The following variables were collected: age, sex, microbiological data, site of infection, presence of comorbidities (diabetes, chronic renal failure, liver failure, solid tumor, malignant hematological disease, heart failure, previous cerebrovascular events, and others), use of invasive therapies (central venous catheter, vesical catheter, mechanical ventilation and hemodialysis), ICU and 28-day all-cause mortality, and ICU and hospital length of stay.

The main outcomes measured were the need for vasopressors during the first 48 hours after inclusion and all-cause 28-day mortality.

This study was approved by the local Ethics Board (CAAE - 0319.0.203.000-11), and all patients or their guardians signed an informed consent form.

### Laboratory assays

Blood samples were obtained at the time of inclusion in the study and on days 3 and 7 of follow-up. Blood samples were centrifuged, and the serum was separated into five aliquots of 0.5mL. These samples were then stored at -80°C.

Circulating C reactive protein (CRP) levels were measured upon inclusion (day 1) and on days 3 and 7 of follow-up, with dry chemistry using Ektachem 950ICR System (Johnson & Johnson Clinical Diagnostics, Inc., Rochester, NY, USA). The detection limit for CRP was 7mg/L. Values above 10mg/dL were considered abnormal.

Interleukin-6 (IL-6), IL-10, high-mobility group box 1 protein (HMGB1) and soluble ST2 protein (sST2) serum levels were assessed in the serum obtained on inclusion and stored at -70°C before later being thawed at room temperature. HMGB1 and sST2 levels were measured through capture ELISA using the HMGB1 Elisa Kit II (IBL International GMB, Hamburg, Germany) and Human ST2/IL-1 R4 Quantikine ELISA Kit (R&D systems, Minneapolis, MN, USA), respectively. Quantification was performed by measuring absorbance at 450nm, and the results are expressed as antigen nanograms per milliliter. IL-6 and IL-10 levels were measured using fluorescent microspheres in flow cytometry through the Cytometric Bead Array (BD Biosciences, Franklin Lakes, NJ, USA) method. All of the procedures were performed according to the corresponding manufacturers' instructions.

### Reactive hyperemia - peripheral arterial tonometry

Microvascular endothelial function was determined using an automated device (EndoPAT2000, Itamar Medical, Caesarea, Israel). The technique has been described elsewhere.^(10)^ Briefly, the cuff was placed on the non-dominant arm, 2cm above the antecubital fossa, and RH-PAT probes were placed on the tip of each index finger. We used the dominant arm in patients monitored with radial artery intra-arterial pressure in the contralateral wrist. After an equilibration period, the baseline pulse amplitude was measured for 5 min. Arterial flow was interrupted on one side for 5 min by inflating the cuff at whichever occlusion pressure would be higher: 200 or 60mmHg above systolic blood pressure. After the 5 min occlusion period, the cuff was deflated to induce reactive hyperemia, and the RH-PAT signals in both hands were recorded for an additional period of 5 min. The contralateral finger was used as a control for systemic changes. The reactive hyperemia index (RHI) is calculated automatically by the RH-PAT device through a formula proposed by the manufacturer. It is defined as the ratio of the post-deflation pulse amplitude 90 to 150 s after cuff release to the average basal pulse amplitude. This result is divided by the corresponding ratio from the control finger and multiplied by a baseline correction factor. The latter intends to adjust the index for the influence of basal vascular tone. Lower RHI values are related to an impairment of the endothelium's vasodilatory response. Two trained investigators (TAB and LVR) executed the RH-PAT exams consecutively (i.e., not as duplicate tests), and one of the coauthors (LCB) performed the quality control for all exams. Reasons to exclude studies were noisy signals, occlusion duration > 5.5 or < 4.5 minutes and breakthrough of the arterial pulse curve during upper-arm occlusion.^(11)^

### Statistical analysis

The categorical variables are presented according to their absolute and relative frequency. Regarding the continuous data, the median and the 25 - 75% interquartile interval (Q1 - Q3) were used for the non-normally distributed variables, whereas the mean and standard deviation (SD) were used for the normally distributed variables. Patients were compared using the chi-squared test or the Fisher exact test and Student's *t* test or Mann-Whitney U test, as appropriate. The results of RHI obtained for the same patients in different time points (i.e., on day 1 and on day 3) were compared using the Wilcoxon signed rank test.

Correlation among continuous variables was evaluated using the Spearman test due to the non-normal nature of these variables. The variables included in these analyses were RHI, Acute Physiology and Chronic Health Evaluation II (APACHE II) score, Sequential Organ Failure Assessment (SOFA), and the circulating molecules (IL-6, IL-10, sST2, HMGB1 and CRP).

A two-tailed test with a significance (p value) of less than 0.05 was set for all of the analyses. All of the data were analyzed using the SPSS statistical package, version 20.1 (SPSS, Chicago, IL).

## RESULTS

**Figure 1:**
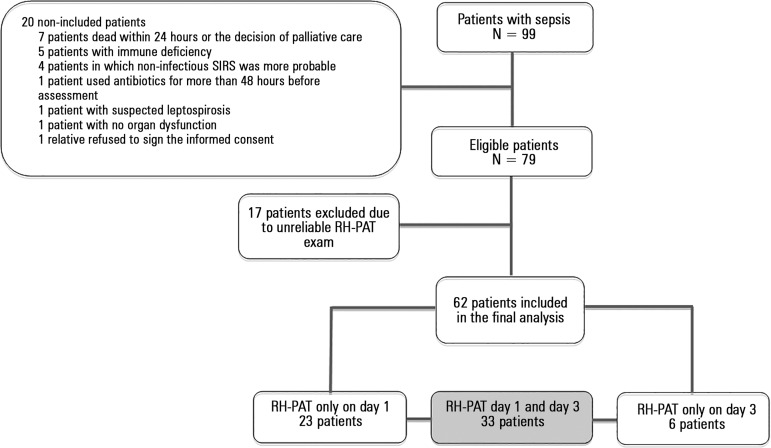
Flowchart of study procedures. SIRS - systemic inflammatory response syndrome; RH-PAT - reactive hyperemia - peripheral arterial tonometry.

Overall, 99 patients with sepsis and with no obvious exclusion criteria were evaluated in the study period, among which 79 underwent the RH-PAT exam within the first 48 hours following inclusion. Seventeen of the 79 patients (21.6%) had the signals obtained in the RH-PAT exams performed within the first 48 hours (either on day 1 or on day 3) considered technically unreliable and were thus excluded from the study (Figure 1). Therefore, the final analysis included 62 individuals with at least one reliable RH-PAT exam. Of note, there was no difference in the proportion of patients with septic shock between the 17 excluded patients (82.4%) and the 62 patients with reliable signs (75.8%) (p = 0.749).

A total of 95 RH-PAT tests were performed in the 62 studied individuals as follows: 56 (90.3%) exams were performed at study inclusion (day 1), and 39 (62.9%) were performed on the third day of follow-up (day 3). Thirty-three (53.2%) out of the 62 patients were evaluated with RH-PAT at both time points, i.e., upon inclusion and on day 3. The reasons for not performing the exams at both time points, i.e., day 1 and day 3 in 29 individuals were the presence of an exclusion criterion on day 3 (14 cases), technical and logistical issues precluding RH-PAT execution on day 1 (6 cases) or on day 3 (5 cases) and death before the third day of follow-up (4 cases). Specifically for day 3, the following exclusion criteria were observed: decision for palliative care (4 cases), psychomotor agitation (8 cases) and low platelets (2 cases). The main characteristics presented by the 62 patients included in the final analysis are shown in table 1. In table 2, the same characteristics are presented in the subgroup of 33 patients with RH-PAT exams performed on day 1 and day 3.

**Table 1:** Main characteristics of the 62 patients included in the study

Characteristics	Overall (N = 62)	Survivors (N = 45)	Non-survivors (N = 17)
Age	51.5 (18.9)	47.4 (15.9)	62.3 (18.4)
Male sex	49 (62.0)	28 (62.2)	11 (64.7)
Type of admission			
Medical	54 (87.1)	40 (88.9)	14 (82.4)
Non-trauma surgery	8 (12.9)	5 (11.1)	3 (17.6)
Comorbidities			
Arterial hypertension	25 (40.3)	17 (37.8)	8 (47.1)
Heart failure	11 (17.7)	7 (15.6)	4 (23.5)
Chronic renal failure	12 (19.4)	7 (15.6)	5 (29.4)
Diabetes	12 (19.4)	9 (20.0)	3 (17.6)
Previous stroke	8 (12.9)	7 (15.6)	1 (5.9)
Chronic coronary disease	10 (16.1)	6 (31.3)	4 (23.5)
Solid neoplasm	8 (12.9)	5 (11.1)	3 (17.6)
Active smoking (last 6 months)	13 (21)	13 (28.9)	0 (0)
Statin use	18 (22.8)	10 (22.2)	4 (23.5)
Known hypercholesterolemia	8 (10.1)	4 (8.8)	1 (5.9)
APACHE II score (first 24 hours)	16 [12 - 20]	14 [9 - 18.5]	19 [17 - 28.5]
SOFA score (first 24 hours)	7 [5 - 9]	6 [5 - 8.5]	9 [6.5 - 12.5]
Confirmed microbiology	37 (59.7)	27 (60.0)	10 (58.8)
Positive blood culture	26 (41.9)	18 (40.0)	8 (47.1)
Site of infection			
Lung	27 (43.5)	20 (44.4)	7 (4.2)
Abdomen	12 (19.4)	8 (17.8)	4 (23.5)
Catheter	7 (11.3)	5 (11.1)	2 (11.8)
Skin and soft tissue	7 (11.3)	4 (8.9)	3 (17.6)
Urine	2 (3.2)	2 (4.4)	0 (0)
Other	7 (11.3)	6 (31.3)	1 (5.9)
Creatinine	1.15 [0.6 - 2.54]	1.5 (1.4)	2.2 (1.6)
Steroids first 48 hours	14 (22.6)	7 (15.6)	7 (41.2)
Inotropics upon inclusion	12 (19.4)	7 (15.6)	5 (29.4)
Vasopressors upon inclusion	47 (75.8)	33 (73.3)	14 (82.3)
Dialysis first 48 hours	13 (21)	6 (13.3)	7 (41.2)
Death in the ICU	14 (22.6)	-	-
Death in the 28 days	17 (27.4)	-	-

APACHE II - Acute Physiology and Chronic Health Evaluation II; SOFA - Sequential Organ Failure Assessment; ICU - intensive care unit. Values are expressed as the mean (SD), number (%) or median [25 - 75%].

**Table 2:** Main characteristics of the 33 patients with reactive hyperemia - peripheral arterial tonometry exams performed on day 1 and on day 3

Characteristics	Overall (N = 33)	Characteristics	Overall (N = 33)
Age	46.5 (18.9)	Post-operative central nervous system	5 (15.1)
Male sex	21 (63.6)	Catheter	4 (12.1)
Type of admission		Skin and soft tissue	2 (6.0)
Medical	29 (87.9)	Urine	1 (3.0)
Non trauma surgery	4 (12.1)	Others	2 (6.0)
Comorbidities		RHI day 1	1.68 [1.40 - 2.04]
Arterial hypertension	14 (42.4)	RHI day 1	1.72 (0.51)
Heart failure	4 (12.1)	RHI day 3	1.61 [1.46 - 2.23]
Chronic renal failure	4 (12.1)	RHI day 3	1.82 (0.51)
Diabetes	5 (15.2)	RHI trend	0.020 [-0.300 - 0.365]
Previous stroke	4 (12.1)	HMGB1 day 1	14.29 [8.02 - 24.55]
Chronic coronary disease	3 (9.1)	IL-6 day 1	277.5 [41.6 - 713.8]
Solid neoplasm	3 (9.1)	IL-10 day 1	3.01 [0.77 - 8.08]
Active smoking (last 6 months)	8 (24.4)	sST2 day 1	1761.3 [925.7 - 3269.0]
Statin use	5 (15.2)	C reactive protein	205.0 [145.5 - 248.5]
Known hypercholesterolemia	3 (9.1)	Creatinine	1.30 [0.5 - 3.30]
APACHE II (first 24 hours)	15 (9-20.5)	Steroids first 72 hours	6 (18.2)
SOFA (first 24 hours)	6 (5-9)	Inotropics first 72 hours	4 (12.1)
Confirmed microbiology	17 (51.5)	Vasopressors first 48 hours	21 (63.6)
Positive blood culture	14 (42.4)	Dialysis first 48 hours	6 (18.2)
Site of infection		Death in the ICU	4 (12.1)
Lung	13 (39.4)	Death in 28 days	6 (18.2)
Abdomen	6 (18.2)		

APACHE II - Acute Physiology and Chronic Health Evaluation II; SOFA - Sequential Organ Failure Assessment; RHI - reactive hyperemia index; HMGB1 - high mobility group box 1 protein; IL-6 - interleukin 6; IL-10 - interleukin 10; sST2 - soluble ST2; ICU - intensive care unit. Values are expressed as the mean (SD), number (%) or median [25 - 75%].

### Reactive hyperemia - peripheral arterial tonometry

RHI values obtained on day 1 and day 3 are shown in table 3. No difference was found in the day 1 RHI values, day 3 RHI values and RHI trend (day 3 - day 1) when the subgroups of patients who required vasopressors or not during the first 48 hours of follow-up were compared (p = 0.179, p = 0.105 and p = 0.868, respectively). Moreover, no significant correlation was observed between RHI values measured on day 1, day 3, or the difference between these values (RHI trend) and scores of organ dysfunction (SOFA), severity (APACHE II) or baseline lactate levels.

**Table 3:** Reactive hyperemia index and biomarker serum levels observed among the studied patients, according to their outcome

Variable	Survivors (N = 45)	Non-survivors (N = 17)	p value
RHI day 1	1.62 [1.30 - 2.03]	1.44 [1.23 - 2.45]	0.203
RHI day 3	1.66 [1.45 - 2.19]	1.53 [1.39 - 1.99]	0.712
RHI trend	-0.080 [-0.320 - 0.340]	0.295 [0.167 - 0.795]	0.045
HMGB1 day 1	15.97 [7.22 - 24.52]	15.23 [9.08 - 23.30]	0.937
IL-6 day 1	316.7 [103.3 - 864.9]	278.1 [195.7 - 1767.7]	0.937
IL-10 day 1	5.04 [1.19 - 11.54]	4.27 [1.66 - 9.11]	0.623
sST2 day 1	1660.5 [976.2 - 2844.4]	2136.8 [1401.3 - 2872.7]	0.222
C reactive protein	223.0 [180.5 - 332.5]	252.5 [198.4 - 357.7]	0.569

RHI - reactive hyperemia index; HMGB1 - high mobility group box 1 protein; IL6 - interleukin 6; IL-10 - interleukin 10; sST2 - soluble ST2. Mann-Whitney test for all comparisons. Values are expressed as median [25 - 75%].

**Figure 2:**
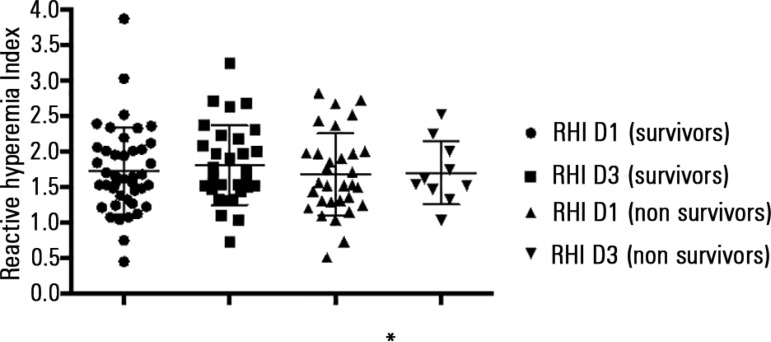
Reactive hyperemia index measured upon inclusion and on the third day of follow-up, according to the 28-day outcome. RHI - reactive hyperemia index; D1 - day 1; D3 - day 3. This analysis was restricted to the subgroup of 33 patients with reactive hyperemia index tested upon the inclusion and on the third day of follow-up. There was a significant increase of the reactive hyperemia index values from inclusion to the third day of follow-up. * p < 0.05.

RHI values measured on day 1 and day 3 were not different among patients who died within 28 days of follow-up compared with the survivors (p = 0.203 and p = 0.712, respectively). In the subgroup of 33 patients with RH-PAT tested on day 1 and day 3, a more expressive increase in RHI values (median increment = 0.295) was observed among non-surviving patients compared with survivors (median reduction = 0.080, p = 0.045) (Figure 2).

### Circulating biomarkers

**Figure 3:**
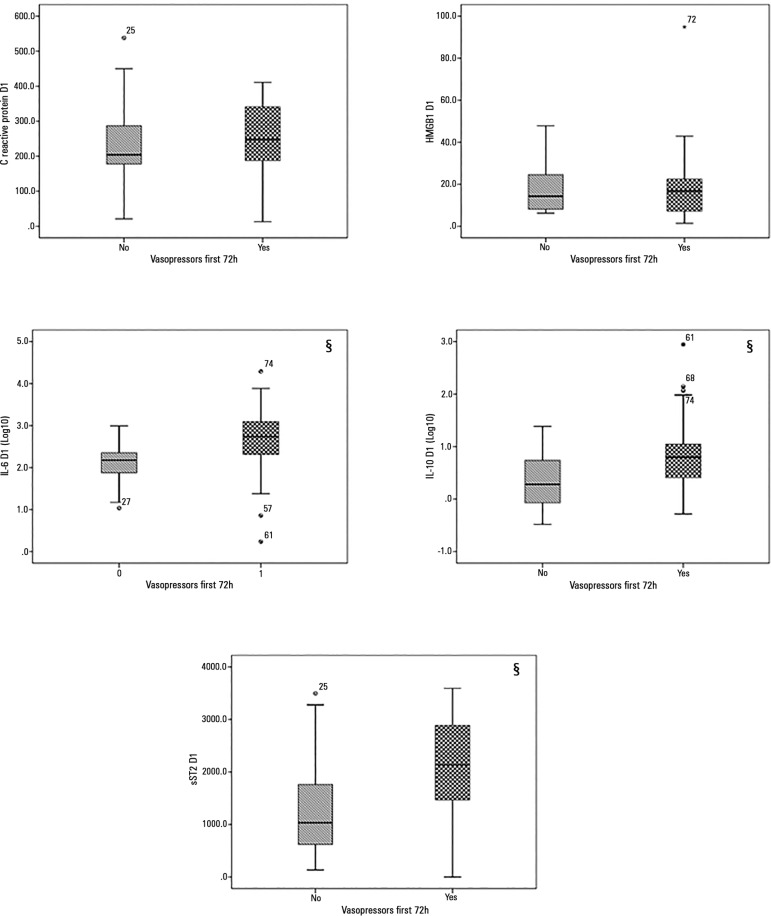
Levels of the tested biomarkers according to the requirement for vasopressors in the studied patients. HMGB1 - High mobility group box 1 protein; IL-6 - interleukin 6; IL-10 - interleukin 10; sST2 - soluble ST2. § p < 0.05.

The median values of circulating CRP and the inflammatory molecules HMGB1, IL-6, IL-10 and sST2 measured upon study inclusion are presented in table 3. None of the tested markers demonstrated significant differences in their median circulating levels between individuals who died within 28 days of follow-up and surviving patients. Regarding the severity of sepsis, we observed that the circulating levels of IL-6 (p = 0.002), IL-10 (p = 0.034) and sST2 (p = 0.020) were significantly higher among patients with septic shock compared with patients with severe sepsis who responded to fluid resuscitation (Figure 3).

A weak negative correlation (r = -0.287) was observed between baseline RHI (day 1) and HMGB1 levels (p = 0.034). No significant correlation was found among the remaining molecules and RHI results.

## DISCUSSION

In this prospective study of septic patients, we found that microvascular endothelial function as assessed by RH-PAT, a non-invasive and user-independent method, was not correlated with the severity of sepsis and was not associated with 28-day all-cause mortality. An unexpected trend of increase (i.e., improvement) in the RHI values was observed among the non-surviving patients. Moreover, excepting a weak negative correlation between HMGB1 levels and RHI values, no correlation was observed among the levels of the tested inflammatory molecules and the RH-PAT results. Lastly, a high proportion of the septic patients initially included in this study had their RH-PAT results rejected due to poor quality, implying a low utility for this method among severely ill patients.

Microcirculatory blood flow and endothelial function can be assessed by different techniques. Biological markers, such as lactate, which represents one classical surrogate of tissue hypoperfusion, are useful to define the severity of disease and to guide initial resuscitation in severe sepsis.^(12)^ Laser Doppler devices, microvideoscopic techniques and nailfold videocapillaroscopy represent interesting tools to assess the microcirculatory state, but all of them face important limitations that preclude their use in the routine management of septic patients.^(13)^ Orthogonal polarization spectral and sidestream darkfield are videomicroscopic techniques and were largely tested in experimental studies. Although interesting clinical studies testing these two devices in septic patients have been reported,^(14,15)^ numerous shortcomings must be overcome before these techniques can be part of the routine arsenal for sepsis management.

Regarding studies assessing endothelial function, Becker et al. compared a group of patients with sepsis (mean APACHE II of 23 ± 7) with healthy controls, and showed that a lower flow-mediated vasodilation (FMD) of the brachial artery, a measurement obtained by a non-invasive ultrasound-based method, was present in the group of septic patients compared with controls.^(16)^ These findings suggest an impairment of compensatory arterial vasodilation in sepsis. Moreover, compared with the survivors, a significantly higher proportion of non-survivors among the septic patients had a decline in the FMD values from the day of inclusion to the third day of follow-up.

Due to its user-independence, easy-to-operate nature, and low time consumption to perform, RH-PAT appears to be an attractive tool to assess endothelial function in the microcirculation of septic patients. Our initial assumption was that the arterial vasodilation response represented by RHI would be inhibited in septic patients, probably due to decreased bioavailability of nitric oxide in a dysfunctional endothelium; we further hypothesized that this inhibition would be proportional to the disease severity and would thus be more pronounced among non-survivors. In fact, the mean RHI value in the studied patients on day 1 (1.71 ± 0.62) was smaller than the RHI value observed by Brant et al., in a recent study of reproducibility conducted in a group of adults (73% were men, mean age approximately 52) who were participants in a cohort about the determinants of cardiovascular diseases in Brazil.^(10)^ In that study, the mean RHI did not vary significantly between two exams performed over an interval of 2 - 6 hours (1.92 ± 0.56 and 1.96 ± 0.58, respectively). The difference observed between Brant's results and ours suggests an impairment of the microcirculation vasodilating response among septic patients or at least in patients with one or more organs with dysfunction.

Davis et al.^(17)^ found results similar to ours, with mean RHI values of 1.57 (CI95%: 1.44 - 1.71) in patients with severe sepsis, a value significantly lower than the value observed among healthy controls.^(9)^ However, in contrast to previous reports, we did not find any significant correlation among RHI values, disease severity and patient outcomes. In their study, Davis et al.^(17)^ demonstrated that the baseline mean RHI observed in a group of 85 septic patients was inversely proportional to the severity of disease (i.e., the presence of shock and APACHE II score). Surprisingly, in our study, patients with poor outcomes presented a slight but significant improvement in RHI from day 1 to day 3. If we assume that our patients were included later in the course of sepsis, our findings could be partially explained by the variation in the activity of endothelial nitric oxide throughout this syndrome.^(18)^ It has been demonstrated that the later stage of sepsis may be characterized by an increase in the production of nitric oxide, which causes diffuse microcirculatory vasodilatation and therefore a fall in blood pressure. This excess in the bioavailability of NO may contribute for the refractory shock observed in the most severe cases of sepsis spectrum patients.^(18)^ Alternatively, this result could be biased because the most severely ill patients may have died before inclusion in the study or before day 3 and therefore were not evaluated by RH-PAT on that day. Regarding this point, it is worth mentioning that the proportion of patients with a length of stay in the ICU of three days or fewer days was similar between survivors and non-survivors (17.8% versus 17.6%, p = 1.00). Finally, because the RHI values measured on day 1 and day 3 were not correlated with mortality, the RHI improvement from inclusion to day 3 among the non-surviving individuals could be the result of chance, and further studies are necessary to confirm these results.

In this study, we also investigated whether baseline circulating levels of CRP and four additional biological markers correlated with RHI values; three of these markers have predominantly pro-inflammatory properties (IL-6, HMGB1 and sST2), and the remaining one is a regulatory cytokine (IL-10). The rationale to measure IL-6 and IL-10 was to better characterize the patient's profile at the time of inclusion in the study, whether inflammatory (IL-6) or anti-inflammatory (IL-10) and whether this state would influence peripheral arterial tonometry. Recently, it was reported that HMGB1 can potentiate the release of the adhesion molecules ICAM-1, VCAM-1 and E-selectin on endothelial membranes, which can be associated with endothelial dysfunction.^(19)^ The broad roles of IL-33 and ST2 in numerous diseases, but mainly in the pathophysiology of cardiovascular diseases, have been demonstrated.^(20)^ We wanted to evaluate whether HMGB1 and soluble ST2 can predict microvascular endothelial function as measured by RH-PAT in septic patients. As presented, only the HMGB1 levels had a significant (weak and negative) correlation with RHI measured on day 1. The meaning of this finding must be investigated in future studies. Interestingly, IL-6, IL-10 and sST2 were significantly higher among patients who needed vasopressors during the first 48 hours of follow-up.

This study has several limitations that must be acknowledged. First, we included a small sample of patients at a single center, limiting the strength of our statistical analysis and the extrapolation of our findings to other settings. Second, we did not include a control group of healthy volunteers or a group of critical care patients without sepsis. To overcome this flaw, we compared the RHI results found in this study with the results published by Brant et al.^(10)^ These authors studied 123 adults with a sex proportion and mean age similar to our patients. Moreover, Brant's study included patients living in the same city where our patients were included. All of these characteristics make these historical controls adequate for the present study. In addition, we were not able to test better and more specific surrogates of endothelial function, such as L-arginine, E-selectin, angiopoietin-2, circulating endothelial cells, among others. Additionally, it should be emphasized that more than 1/5 of septic patients initially included in this study had to be excluded from the final analysis because their RH-PAT results were not reliable. RH-PAT has been described as a method to be used in a controlled environment^(21)^ where adequate light, appropriate temperature and the patient's cooperation are necessary to obtain valid results. Moreover, the use of medications could have influenced the results. The proportion of unreliable results cited above makes us reticent about the validity of this method to assess microvascular endothelial function in ICU patients. Last, we were not able to specify the exact number of hours that elapsed between the diagnosis of sepsis and the RH-PAT exams.

## CONCLUSION

In this study of septic patients presenting with at least one organ in dysfunction, we found that reactive hyperemia - peripheral arterial tonometry results were unable to distinguish individuals more severely ill from those with less severe disease. Furthermore, this exam did not identify individuals with poor outcomes among the studied patients. Reactive hyperemia - peripheral arterial tonometry results on day 1 correlated negatively with high-mobility group box 1 protein levels measured upon inclusion, and this finding deserves more investigation. In addition to its poor prognostic ability, reactive hyperemia - peripheral arterial tonometry also proved to be a tool of limited use in intensive care patients, showing unreliable results in up to one-fifth of exams.
